# Sphingolipids and physical function in the Atherosclerosis Risk in Communities (ARIC) study

**DOI:** 10.1038/s41598-020-80929-3

**Published:** 2021-01-13

**Authors:** Danni Li, Aniqa B. Alam, Fang Yu, Anna Kucharska-Newton, B. Gwen Windham, Alvaro Alonso

**Affiliations:** 1 Department of Lab Medicine and Pathology, University of Minnesota, 420 Delaware Street SE, MMC 609, Minneapolis, MN 55455 USA; 2 Department of Epidemiology, Rollins School of Public Health, Emory University, Atlanta, GA 30322 USA; 3 School of Nursing, University of Minnesota, Minneapolis, MN 55455 USA; 4 Department of Epidemiology, Gillings School of Global Public Health, University of North Carolina, Chapel Hill, NC 27514 USA; 5 Department of Medicine, University of Mississippi Medical Center, Jackson, MS 39210 USA

**Keywords:** Biochemistry, Biomarkers

## Abstract

Long-chain sphingomyelins (SMs) may play an important role in the stability of myelin sheath underlying physical function. The objective of this study was to examine the cross-sectional and longitudinal associations of long-chain SMs [SM (41:1), SM (41:2), SM (43:1)] and ceramides [Cer (41:1) and Cer (43:1)] with physical function in the Atherosclerosis Risk in Communities (ARIC) study. Plasma concentrations of SM (41:1), SM (41:2), SM (43:1), Cer (41:1) and Cer (43:1) were measured in 389 ARIC participants in 2011–13. Physical function was assessed by grip strength, Short Physical Performance Battery (SPPB), 4-m walking speed at both 2011–13 and 2016–17, and the modified Rosow-Breslau questionnaire in 2016–2017. Multivariable linear and logistic regression analyses were performed, controlling for demographic and clinical confounders. In cross-sectional analyses, plasma concentrations of SM 41:1 were positively associated with SPPB score (β-coefficients [95% confidence internal]: 0.33 [0.02, 0.63] per 1 standard deviation [SD] increase in log-transformed concentration, p value 0.04), 4-m walking speed (0.042 m/s [0.01, 0.07], p value 0.003), and negatively with self-reported disability (odds ratio = 0.73 [0.65, 0.82], p value < 0.0001). Plasma concentrations of the five metabolites examined were not significantly associated with longitudinal changes in physical function or incidence of poor mobility. In older adults, plasma concentrations of long-chain SM 41:1 were cross-sectionally positively associated with physical function.

## Introduction

Physical function decline affects nearly 40% of Americans older than 65 years of age^1^ and leads to many adverse health outcomes^2–4^. Physical function decline may result from adverse physiological perturbations, such as insulin resistance or inflammation^5^. In addition, demyelinating diseases have been increasingly linked to mobility impairments (e.g., multiple sclerosis)^6–8^. In our previous reports, based on findings from the Atherosclerosis Risk in Communities (ARIC) study^9,10^, we observed that higher plasma concentrations of three hydroxysphingomyelins (SM[OH]s) [SM (OH) C22:1, SM (OH) C22:2, and SM (OH) C24:1] were positively associated with physical function cross-sectionally^10^ and total brain volume^9^. A limitation of the previous studies was the use of Biocrates p180 kits (Biocrates Life Science AG, Innsbruck, Austria) and a triple quadrupole mass spectrometer for targeted lipidomic analysis, which cannot discern lipid molecules with a very small mass difference (i.e., less than 0.05 Da) such as SM (OH) C22:1 (M.W. 801.6485 Da) and long-chain SM 41:1 (M.W. 801.6844 Da). Based on an internal study conducted by the company that used Biocrate AbsoluteIDQ^®^ p400 HR kits and a mass spectrometer that has a better mass discerning ability such as Thermo Q Exactive, these three SM(OH)s should have been annotated as long-chain SMs (SM 41:1, SM 41:2, and SM 43:1) instead^11^. Therefore, the question is whether these long-chain SMs (SM 41:1, SM 41:2, and SM 43:1) are associated with physical function.

Sphingomyelins belong to the sphingolipid family, which is a class of lipids containing sphingosine. Other sphingolipids include ceramides and glycosphingolipids. Metabolisms of sphingolipids are regulated by various enzymes and fluxes of different metabolites including amino acid serine and lipid palmitate^12^. Sphingolipids including sphingomyelins are involved in many cellular process such as growth regulation, cell migration, adhesion, apoptosis, and senescence and inflammatory response^12^. Both SM (OH)s and long-chain SMs are abundant in the brain and peripheral nerves^13^. SM (OH)s play important roles in the long-term stability of myelin sheath, lack of which can lead to late onset of demyelination and neurodegeneration^14^ and physical impairment. Long-chain SMs are breakdown products of SM(OH)s, an enzymatic process used to control the levels of SM (OH)s^15^. Long-chain SMs can be synthesized de novo from long-chain ceramides. The primary objective of this study was to examine the cross-sectional and longitudinal associations of three long-chain SMs [SM (41:1), SM (41:2) and SM (43:1)] and two ceramides (Cer [41:1] and Cer [43:1]) with physical function in older adults in the Atherosclerosis Risk in Communities (ARIC) study. In addition, we used self-reported disability (based on self-reported functional status score) and incident poor mobility as secondary outcomes in the cross-sectional analysis and longitudinal analysis, respectively, and explored other plasma SLs in hypothesis generating analysis.

## Methods

### Study population

The ARIC study is a longitudinal cohort study of 15,792 persons recruited in 1987–1989 from four United States communities: Minneapolis suburbs, MN; Forsyth County, NC; Washington County, MD; and Jackson, MS^16^. Seven study visits have been completed and this study includes visit 5/ARIC Neurocognitive Study (visit 5/ARIC-NCS, 2011–2013) and visit 6 (2016–2017). The ARIC Study and ARIC-NSC protocols were approved by the Institutional Review Board (IRB) of each participating center, and informed consent was obtained from participants at each study visit. The present study was reviewed and approved by the University of Minnesota IRB and informed consent was obtained for the present study.

Three hundred eighty-nine participants were randomly selected from ARIC visit 5 based on the following inclusion criteria: (i). “frozen never-thawed” fasting plasma samples from ARIC visit 5 were available; (ii) physical function data (grip strength, Short Physical Performance Battery [SPPB] score, 4-m walking speed) were available from both ARIC visits 5 and 6; (iii). cognition data were available from both visits 5 and 6. We also excluded 383 participants whom we had studied previously^10^, because we wanted to validate our previous findings on the cross-sectional association between SM (OH)s (now known as long-chain SMs) and physical function in an independent sample.

### Biocrate AbsoluteIDQ^®^ p400 HR kits

Biocrates AbsoluteIDQ^®^ p400 HR kits (Biocrates Life Science AG, Innsbruck, Austria) coupled with a Thermo Q Exactive mass spectrometer was used to measure plasma concentrations of 14 Cholesteryl Esters (CE) and 39 SL ([SM (n = 30) and Ceramides (n = 9)] in a flow injection method. We examined 5 SLs [SM (41:1), SM (41:2), SM (43:1), Cer (41:1) and Cer (43:1)] in the primary hypothesis and the rest of the 14 CE and 34 SLs in a hypothesis generating analysis. The plasma samples were analyzed across 6 batches (each p400 HR kit can measure up to 80 samples). MedIDQ software (Biocrates, Innsbruck, Austria) was used to normalize data based on quality control samples. All the methods were carried out in accordance with relevant guidelines and regulations.

### Outcomes

This study included 3 physical function outcomes measured at both ARIC visits 5 and 6: grip strength, SPPB and 4-m walking speed. Details of the methods were described previously^10^. In summary, grip strength (kg) was assessed in the participant’s preferred hand with the better score from 2 trials used in the analysis^17,18^. Lower extremity physical function was assessed using the SPPB^2^ which is composed of 3 tasks: (i) repeated chair stands; (ii) balance (standing, semi-tandem, tandem); and (iii) a 4-m usual-paced walk (m/s)^18^. Walking aids were allowed for the walking task only. Scores of 0–4 were assigned for each task, based on timed performance of the 3 tasks using established age-based thresholds, then summed for a final score of 0–12. Higher scores represent better function. The 4-m walking speed was evaluated separately from the SPPB because it is considered the sixth vital sign of health in older adults and is a sensitive predictor of mortality and frailty^19–21^. Change in physical function was calculated for each function measure over a mean (SD) of 4.85 (0.58) years (between ARIC visits 5 and 6).

Self-reported disability was examined as a secondary outcome in a cross-sectional analysis only, which is based on self-reported functional status assessed as a summation of the 4 questions from a modified Rosow-Breslau questionnaire administrated in 2016–2017 over the phone, 5 years after the ARIC visit 5^22,23^. The 4 questions were: (1) Are you able to do your usual activities, such as work around the house or recreation? (2) Are you able to walk half a mile without help? That's about 8 ordinary blocks. (3) Are you able to walk up and down stairs without help? (4) Are you able to do heavy work around the house, like shoveling snow or washing windows, walls or floors, without help? Self-reported functional status score ranges 0–4, with higher values representing higher function. Participants answering no to any of the questions were considered self-reported disabled (i.e., self-reported functional status score of 3 or less)^24^. After excluding missing observations (i.e., responses to any of the questions was missing), there were 359 observations left for this secondary analysis.

Additional analysis examined a longitudinal decline in mobility from the ARIC visit 5 to visit 6. We included 291 from the original 389 participants that presented with “normal” mobility at visit 5, defined as an SPPB ≥ 7 and walking speed ≥ 0.8 m/s. Incident poor mobility was defined as either an SPPB < 7 or walking speed < 0.8 m/s at visit 6^25^.

### Covariates

Education level (high school graduate or above/not high school graduate), sex (male/female), and race-site (Forsyth/White, Forsyth/Black, Washington/White, Minneapolis/White, Jackson/Black) were self-reported by the participants at the ARIC study baseline visit (i.e., visit 1) in 1987–1989. The genotyping of APOE polymorphisms was performed using the TaqMan assay (Applied Biosystems, Foster City, CA) during the ARIC visit 3 in 1993–1995^26^. The rest of covariate information below was obtained at the ARIC visit 5 in 2011–2013: smoking status (current smoker/former or never smoker) and drinking status (current drinker/former or never drinker) was self-reported; participants were asked to bring their medications, which were then recorded; use of lipid-lowering medications were considered yes if used in past 4 weeks; body mass index (BMI) was defined as weight in kilograms divided by the square of height in meters measured with the participant wearing light clothing; a sports index for physical activity was calculated based on the number of times per month that participants engaged in vigorous, moderate, or light physical activity and scored 1–5 as previously described^27^; coronary heart disease, stroke and heart failure were defined according to published criteria^28,29^; diabetes was defined as a fasting blood glucose ≥ 126 mg/dL, non-fasting glucose ≥ 200 mg/dL, self-reported physician-diagnosis of diabetes or use of anti-diabetic medication; hypertension was defined as systolic ≥ 140 mmHg or diastolic ≥ 90 mmHg or on hypertension medications; blood levels of total cholesterol, HDL cholesterol, and triglycerides were measured using standard methods^30^. The Digit Symbol Substitution test (DSST) (z-score) is a test of executive function, sustained attention, and processing speed. Participants translated symbols to numbers using a key; the score was the number of correct translations within 90 s^31^. Low DSST score is a risk factor for physical function decline^32^.

### Statistical analysis

Visit 5 characteristics of “eligible and included” and “eligible but excluded and ineligible” were expressed as frequency and % for categorical variables and mean (SD) for continuous variables, which were compared using Wald chi-squared test and using two sided t-test assuming equal variance, respectively. All metabolite concentrations were log-transformed and modeled as z scores. The association between each metabolite and physical function was estimated using multivariable linear regression models. We adjusted for variables previously associated with plasma concentrations of SMs (i.e., age and BMI)^33^, with physical function decline, or with both (i.e., prevalence of diabetes, coronary heart disease, stroke, use of hypertension medication, lipid lowering medications, concentration of total cholesterol or HDL cholesterol, cognitive decline). We considered cognitive function related covariates (i.e., APOE4 genotype and cognition) because cognitive function decline is associated with physical function decline^34^.

Cross-sectional analysis was performed with self-reported functional status scores as discrete data and did not find any significant associations. The association of each metabolite with self-reported disability (i.e., self-reported functional status score of 3 or less) was estimated using logistic regression models, with the outcome modeled as either reporting disability or no disability. The association of each metabolite with incident poor mobility by visit 6 was estimated using logistic regression models, with the outcome modeled as either normal or poor mobility based on the thresholds defined above. Missing values were imputed using multiple imputation by chained equations (MICE) assuming missing, at random (i.e., the probability of the variable being missing is not dependent on the true value of the variable). The MICE method uses the fully conditional specification (FCS) to account for both binary and discriminant variables and implement conditional distributions for each imputed variable, which were calculated based on visit 5 covariates through Proc MI in SAS 9.4. All models were weighted by the inverse probability of being selected to the study. These selection probabilities were calculated using logistic regression modeling with selection to the study as the outcome variable and age, sex, race-center, education, hypertension, previous coronary heart disease, previous stroke, and cognitive status as the predictor variables. We included cognitive status as a predictor variable because it accounts for potential impediments to show up at the later visit which would affect the likelihood of being included in the study. In the hypothesis-generating analysis, we estimated the association of an additional 14 CEs and 35 SLs with measures of physical function and attempted to correct for multiple testing by calculating false discovery rates (FDR) using the Benjamini and Hochberg method^35^. FDR adjusted p values of less than 0.05 indicate significance. In addition, we performed Principle Component Analysis (PCA) on all the metabolites except SM 31:0 which was excluded due to significant missing values. For the rest of the metabolites, missing values were imputed. The PCA analysis generated 4 factors which explained 50% of the total variance. Next, we performed multivariable linear regressions using the 4 factors for each of the outcomes. All analyses were performed using SAS 9.4 (Cary, NC; SAS Institute Inc).

## Results

This study included 389 ARIC Visit 5 participants with mean (standard deviation [SD]) age of 74.7 (4.8) years, of which 49.9% were female and 28.3% African American (Table 1). Compared to remaining eligible but excluded participants and ineligible participants (Supplementary Table 1), the study participants were younger, more likely to be male or African Americans, had slightly higher BMI, better grip strength and SPPB score.

**Table 1:** Visit 5 characteristics of eligible and included and eligible but excluded and ineligible Atherosclerosis Risk in Communities (ARIC) Study participants (2011–2013).

	Eligible and included (n = 389)	Eligible but excluded and ineligible (n = 6149)	P-value*
Age, years	74.7 (4.8)	75.9 (5.3)	< 0.0001
Female, %	194 (49.9)	3641 (59.4)	0.0002
Black, %	110 (28.3)	1434 (23.4)	0.03
Body mass index, kg/m^2^	29.4 (6.0)	28.7 (5.8)	0.03
Diabetes, %	124 (31.9)	2077 (33.9)	0.42
Hypertension, %	292(75.1)	4523 (74.9)	0.94
On lipid lowering medication, %	205 (52.4)	3420 (56.1)	0.15
Total cholesterol, mg/dL	182.1 (39.8)	181.3 (42.2)	0.71
Executive z-score	− 0.42 (0.81)	− 0.48 (0.85)	0.21
Previous stroke, %	11 (2.8)	262 (4.3)	0.17
Grip strength (kg)	31.2 (10.7)	29.0 (10.3)	< 0.0001
SPPB score (0–12 scale)	9.7 (2.2)	9.2 (2.6)	0.0004
4-m walking speed (m/s)	0.95 (0.22)	0.93 (0.23)	0.06

Data are shown as frequency (percentage) for categorical variables and as mean (standard deviation) for continuous variables.

*All categorical p-values calculated using Wald chi-squared test and continuous using t-test.

**Figure 1:**
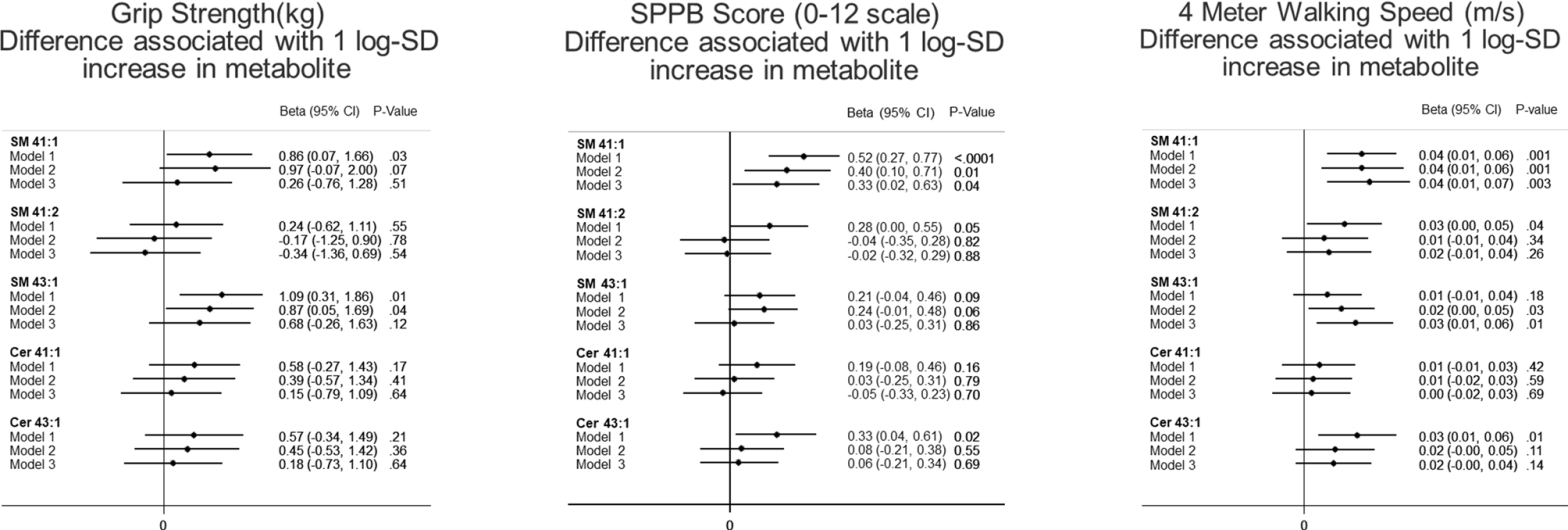
Cross-sectional weighted associations of sphingomyelins and ceramides with physical function in ARIC visit 5. Multiple linear regression model was used to determine β-coefficients [95% confidence internal] between each metabolite (log-transformed concentration modeled in z score) and physical function. Model 1 covariates: age, sex, race-site, and batch effects. Model 2 covariates: model 1, plus body mass index, diabetes status, hypertension status, use of lipid lowering medication, total cholesterol, Digit Symbol Substitution test z-score, and prevalent stroke at the ARIC visit 5. Model 3 covariates: model 2, plus education, presence of an APOE4 allele, Mini-mental state examination (MMSE) score, sports index, previous heart failure, previous coronary heart disease, CES depression score, HDL cholesterol, triglyceride levels, SF bodily pain score, current smoking status, and current drinking status.

Among the five SLs in the primary analysis, we observed that SM 41:1 was cross-sectionally in positive association with better SPPB score and 4-m walking speed in all three models (Fig. 1) (β-coefficients [95% confidence internal] and their p values in Models 1, 2, 3 were 0.52 [0.27, 0.77], 0.40 [0.10, 0.71], 0.33 [0.02, 0.63], and < 0.001, 0.01, and 0.04 respectively, per 1 SD increase in log-transformed concentration for SPPB score and 0.04 m/s [0.01, 0.06], 0.04 m/s [0.01, 0.06], 0.042 m/s [0.01; 0.07] and 0.001, 0.001, and 0.003, respectively, per 1 SD increase in log-transformed concentration for walking speed). The strength of the associations met the minimal clinically meaningful differences (0.27–0.55 units) for SPPB score and approached thresholds for small meaningful clinical differences (0.04–0.06 m/s) for walking speed^36^.

**Figure 2:**
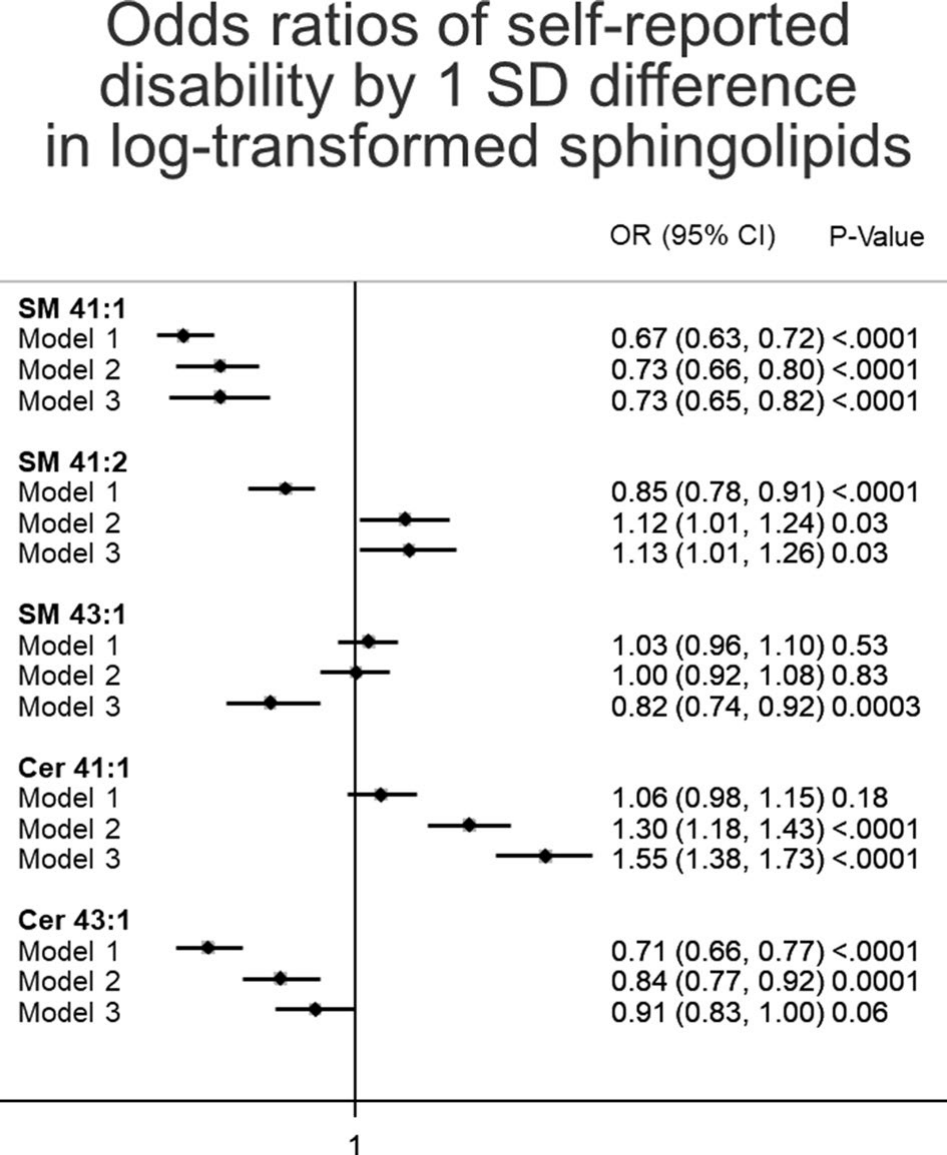
Odds ratios of self-reported disability by 1 SD difference in log-transformed sphingolipids, ARIC 2011–2017. The association of each metabolite with self-reported disability was estimated using logistic regression models, with the outcome modeled as either reporting disability or no disability. Odds ratios less than 1 indicate a protective effect. Number of individuals reporting disability was 103 (n = 359). *SD* standard deviation, *OR* odds ratio, *CI* confidence interval, *SM* sphingomyelins, *Cer* ceramides. Model 1: logistic regression adjusted for age, sex, race-site, and batch effects. Model 2: as model 1, plus additional adjustment for body mass index, diabetes status, hypertension status, use of lipid lowering medication, total cholesterol, Digit Symbol Substitution test z-score, and prevalent stroke at the ARIC visit 5. Model 3: as model 2, plus additional adjustment for education, presence of an APOE4 allele, Mini-mental state examination (MMSE) score, sports index, previous heart failure, previous coronary heart disease, CES depression score, HDL cholesterol, triglyceride levels, SF bodily pain score, current smoking status, and current drinking status.

SM 41:1 was also cross-sectionally associated with lower odd ratios of self-reported disability (Fig. 2) (odd ratios and their p values in all three models were 0.67 [0.63, 0.72], 0.73 [0.66, 0.80], and 0.73 [0.65, 0.82] and < 0.0001, < 0.0001, < 0.0001, respectively). Interestingly, odd ratios of Cer41:1 and Cer 43:1 with self-reported disability increased after covariate adjustments For Cer 41:1, its odd ratio with self-reported disability in Model 3 was 1.55 [1.38, 1.73], p value < 0.0001, suggesting the association of higher Cer 41:1 with worse self-reported disability. None of the other four SLs demonstrated any significant associations with self-reported disability in all models (Fig. 2).

**Figure 3:**
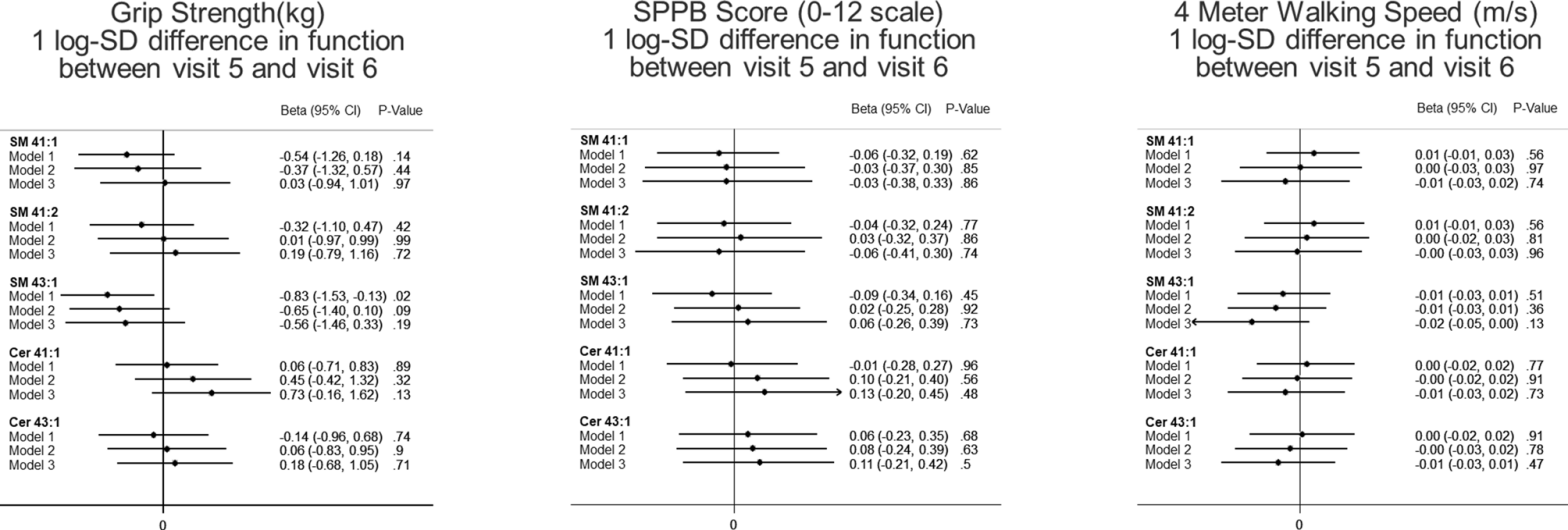
Longitudinal weighted associations of sphingomyelins and ceramides with change in physical function between ARIC visit 5 and 6. Multiple linear regression model was used to determine β-coefficients [95% confidence internal] between each metabolite (log-transformed concentration modeled in z score) and changes in physical function. Model 1 covariates: age, sex, race-site, and batch effects. Model 2 covariates: model 1, plus body mass index, diabetes status, hypertension status, use of lipid lowering medication, total cholesterol, Digit Symbol Substitution test z-score, and prevalent stroke at the ARIC visit 5. Model 3 covariates: model 2, plus education, presence of an APOE4 allele, Mini-mental state examination (MMSE) score, sports index, previous heart failure, previous coronary heart disease, CES depression score, HDL cholesterol, triglyceride levels, SF bodily pain score, current smoking status, and current drinking status.

**Figure 4:**
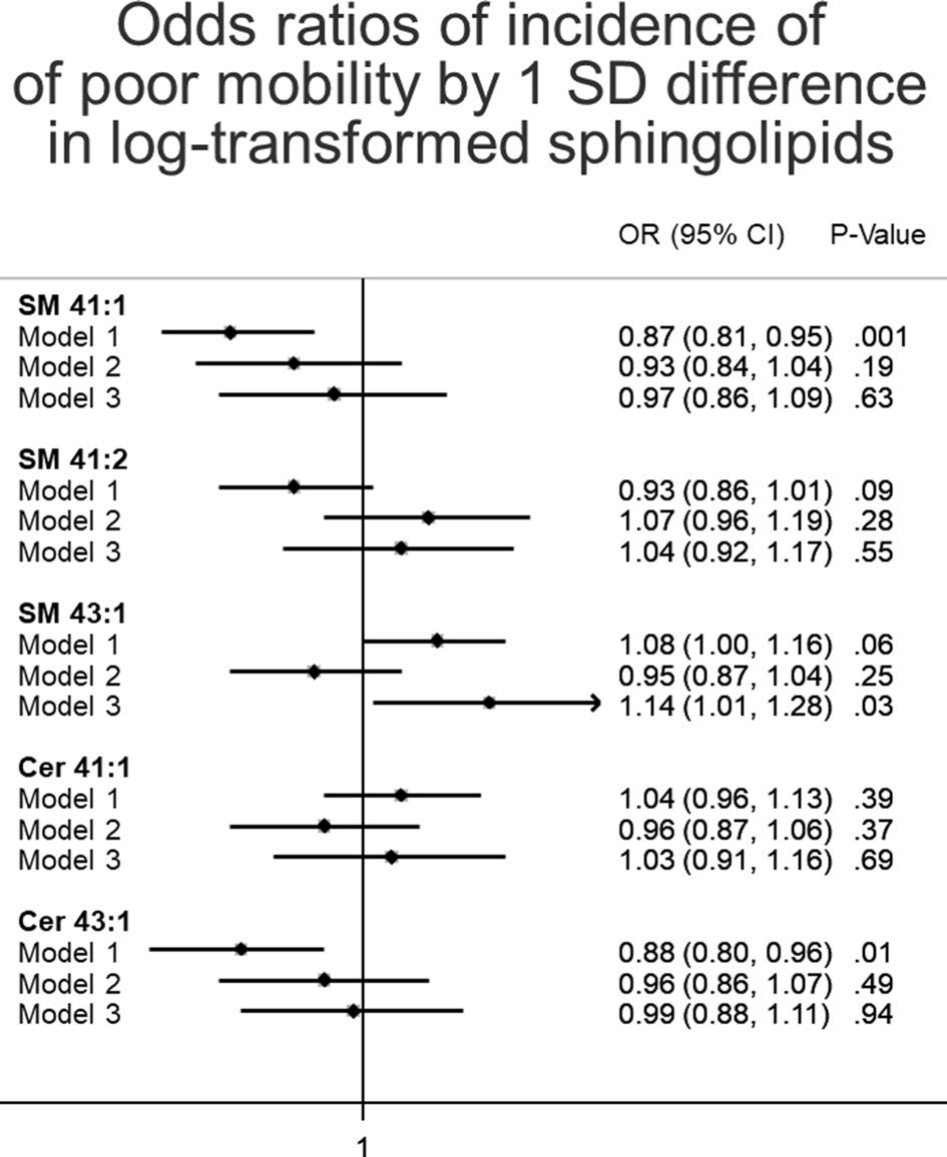
Odds ratios of incident of poor mobility by 1 SD difference in log-transformed sphingolipids, ARIC 2011–2017. The number of "normal" participants with a SPPB ≥ 7 and 4-m walking speed ≥ 0.8 m/s at visit 5 is 291; the number of participants with incident poor mobility (SPPB < 7 or walking speed < 0.8 m/s) is n = 80. The association of each metabolite with incident poor mobility by visit 6 was estimated using logistic regression models, with the outcome modeled as either normal or poor mobility based on the thresholds defined above. Model 1: logistic regression adjusted for age, sex, race-site, and batch effects. Model 2: as model 1, plus additional adjustment for body mass index, diabetes status, hypertension status, use of lipid lowering medication, total cholesterol, Digit Symbol Substitution test z-score, and prevalent stroke at the ARIC visit 5. Model 3: as model 2, plus additional adjustment for education, presence of an APOE4 allele, Mini-mental state examination (MMSE) score, sports index, previous heart failure, previous coronary heart disease, CES depression score, HDL cholesterol, triglyceride levels, SF bodily pain score, current smoking status, and current drinking status.

In the longitudinal analyses, SM 43:1 were associated with change in physical function (Fig. 3) or incident of poor mobility (Fig. 4). In the hypothesis generating analyses, none of the metabolites included were significantly associated with physical function cross-sectionally or longitudinally (Supplementary Figs. 1, 2).

Furthermore, we performed PCA analysis generated the 4 factors that explained more than 50% variance (Supplementary Table 1). Among the 4 factors, factor 1 was cross-sectionally in positive association with better SPPB score and 4-m walking speed in Model 1, although the associations were attenuated and no longer significant in Models 2 and 3 (Supplementary Table 3).

## Discussion

This study examined the cross-sectional and longitudinal associations of sphingolipids SM (41:1), SM (41:2) SM (43:1) and ceramides Cer (41:1) and Cer (43:1) with physical function in older adults. The major study findings were that plasma concentrations of SM 41:1 were significantly in positive association with higher SPPB score, faster 4-m walking speed, and lower self-reported disability. This study did not find any significant longitudinal associations of the five SLs with a 5-year change in objectively assessed physical function or incident poor mobility.

How plasma concentration of SM 41:1 may be cross-sectionally associated with better physical function remains speculative. Long-chain SMs are alpha oxidation products of SM (OH)s^15^, which play an important role in the long-term stability of myelin sheath^14^. The observed positive cross-sectional association between SM 41:1 and physical function is consistent with the observations that SM 41:1 is abundant in nerve cell axons^13^. This study did not find a cross-sectional association of SM 41:2 or SM 43:1 with physical function, which may be due to different effects of the fatty acid moiety of the SM molecules^37,38^. For example, Bergman et al. found that SM C18:0 was positively related to risk of diabetes whereas SM C14:0, C22:3 and C24:4 were negatively related to diabetes risk^37^. Hanamatsu et al. found that SM C18:0 and 24:0 significantly correlated with the parameters for obesity and insulin resistance; however, SM containing unsaturated acyl chains were not associated with these parameters^38^. It is plausible that sphingomyelin chain length has different associations with health-related outcomes and disease via their effects on protein distribution in plasma memberane^39^. Cer 41:1 was not significantly associated with physical function, suggesting the cross-sectional association of SM 41:1 with physical function may be independent of Cer 41:1.

The reasons for the null findings on the longitudinal associations of selected sphingolipids with physical function may be multiple. First, there is growing evidence that physical function decline may result from dysregulation of multiple physiological systems, so the independent contribution of any 1 system may be relatively small, if any. Second, the study follow-up time may not be long enough. However, the mean (SD) change in SPPB score was − 1.1 (2.4), larger than the smallest meaningful change of 0.27–0.55 in SPPB in older adults^36^. Lastly, plasma concentrations of long-chain SMs may not be independent of the physiological systems that contribute to physical function decline. For example, plasma concentrations of SMs are influenced by chronic inflammatory disease, cardiovascular disease, diabetes, and lack of exercise, all of which can be associated with physical functional decline^22,40–44^.

Strengths of this study include the use of Biocrates HR p400 kit coupled with a mass spectrometry with high mass accuracy (i.e., Thermo Q Exactive), which gives accurate identification of SM 41:1, SM 41:2, SM 43:1, Cer 41:1 and Cer 43:1. However, despite the improvement, Biocrates HR p400 kit still measures a sum signal of all isomeric compounds, preventing the identification of the main plasma isomer of SM 41:1, SM 41:2, and SM 43:1. Future work is needed to further improvement the ability to discern molecular isomers. Additional strengths include the use of the ARIC cohort, which provides excellent phenotyping, repeated measurements of physical function, racial diversity, and stored samples. Furthermore, the study includes both interviewer-assessed physical function (e.g., 4-m walking speed) and self-reported functional status. This study is limited by its small sample size, therefore the study may have been under-powered to detect significant associations. Another limitation is the selection of participants with non-missing physical function and cognitive function (except 30 missing self-reported function status), which have led to a sample with better physical function at baseline and less physical function decline over time compared to those who were not eligible for the study.

## Conclusions

This study reported positive cross-sectional study findings on SM 41:1 and physical function. However, we did not observe any significant associations of plasma levels of selected sphingolipids with longitudinal changes in physical function in older adults.

## Supplementary Information

Supplementary Information.
