# Supplementary material for: Sphingolipids and physical function in the Atherosclerosis Risk in Communities (ARIC) study
Source: Sci Rep. 2021 Jan 13;11:1169. doi: 10.1038/s41598-020-80929-3 (PMC7806657; doi:10.1038/s41598-020-80929-3)

**Sphingolipids and physical function in the Atherosclerosis Risk in Communities (ARIC) study**

Danni Li, PhD, Aniqa B. Alam, BS, Fang Yu, PhD, Anna Kucharska-Newton PhD, MPH, B. Gwen Windham,MD Alvaro Alonso, MD, PhD

**Supplementary Table 1.** Visit 5 characteristics of eligible and included, eligible but excluded, and ineligible Atherosclerosis Risk in Communities (ARIC) Study participants (2011 to 2013).

|  | **Eligible and included (n = 389)** | **Eligible, but excluded (n = 2,655)†** | **Ineligible and excluded (n = 3,494)‡** | **P-value*** |  |
| --- | --- | --- | --- | --- | --- |
| **Follow-up, years** | 4.85 (0.58) | 4.87 (0.60) | 4.68 (0.68) | 0.56 |  |
| **Age, years** | 74.7 (4.8) | 74.3 (4.5) | 77.1 (5.5) | <.0001 |  |
| **Female, %** | 49.9 | 59.0 | 59.7 | 0.001 |  |
| **Black, %** | 28.3 | 18.8 | 26.8 | <.0001 |  |
| **Body mass index, kg/m2** | 29.4 (6.0) | 28.6 (5.3) | 28.7 (6.1) | 0.05 |  |
| **Diabetes, %** | 31.9 | 28.7 | 37.8 | <.0001 |  |
| **Hypertension, %** | 75.1 | 70.7 | 78.0 | <.0001 |  |
| **On lipid lowering medication, %** | 52.7 | 55.5 | 56.7 | 0.27 |  |
| **Total cholesterol, mg/dL** | 182.1 (39.8) | 182.0 (40.5) | 180.8 (42.2) | 0.20 |  |
| **Executive z-score** | -0.42 (0.80) | -0.24 (0.77) | -0.69 (0.86) | <.0001 |  |
| **Previous stroke, %** | 2.8 | 2.7 | 5.5 | <.0001 |  |
| **Mini–Mental State Examination (MMSE)** | 27.6 (2.3) | 28.1 (1.9) | 26.5 (3.9) | <.0001 |  |
| **CES-Depression scale** | 2.8 (2.8) | 2.8 (2.8) | 3.5 (3.3) | <.0001 |  |
| **SF-12 Bodily Pain scale** | 80.3 (23.8) | 79.9 (23.8) | 74.5 (27.5) | <.0001 |  |
| **High school graduate or higher, %** | 89.2 | 89.7 | 80.7 | <.0001 |  |
| **HDL Cholesterol, mg/dL** | 51.1 (13.6) | 52.5 (13.7) | 52.0 (14.3) | 0.05 |  |
| **Triglycerides, mg/dL** | 127.1 (56.3) | 124.8 (59.1) | 126.7 (68.5) | 0.29 |  |
| **hsCRP, mg/L** | 3.9 (5.0) | 3.5 (6.9) | 5.0 (10.2) | <.0001 |  |
| **APOE-4 allele** | 26.2 | 28.1 | 30.3 | 0.07 |  |
| **Current smoker** | 7.5 | 4.8 | 6.3 | 0.02 |  |
| **Current drinker** | 52.7 | 53.2 | 44.6 | <.0001 |  |
| **Sports Index (0-4)** | 2.7 (0.8) | 2.7 (0.8) | 2.5 (0.8) | <.0001 |  |
| **Prevalent coronary heart disease** | 8.5 | 13.8 | 18.7 | <.0001 |  |
| **Prevalent heart failure** | 9.3 | 8.5 | 18.5 | <.0001 |  |
| **Grip strength (kg) at V5** | 31.3 (10.6) | 30.0 (10.3) | 28.1 (10.3) | <.0001 |  |
| **SPPB score (0-12 scale) at V5** | 9.7 (2.2) | 9.9 (2.0) | 8.6 (2.9) | <.0001 |  |
| **4 meter walking speed (m/s) at V5** | 0.95 (0.22) | 0.98 (0.21) | 0.88 (0.23) | <.0001 |  |
| **Change in grip strength between V5 and V6** | -3.6 (6.4) | -3.0 (6.1) | -3.9 (6.1) | 0.02 |  |
| **Change in SPPB score between V5 and V6** | -1.1 (2.4) | -0.9 (2.1) | -2.6 (3.1) | <.0001 |  |
| **Change in 4 meter walking speed between V5 and V6** | -0.11 (0.18) | -0.11 (0.19) | -0.13 (0.17) | 0.71 |  |
| **Self-reported physical functioning (0-4 scale)** | 3.50 (0.95) | 3.48 (0.96) | 2.92 (1.34) | <.0001 |  |
| *All categorical p-values calculated using Wald chi-squared test and continuous using analysis of variance (ANOVA). | | | | |  |
| †Excluded due to lack of plasma data. | | | | |  |
| ‡Ineligibility based on lack of cognition data or physical functioning data at visits 5 and/or 6, Asian or Native American descent, low race-center counts, or missing consent. | | | | |  |
|  |  |  |  |  |  |

**Supplementary Table 2.** Summary statistics for SM (41:1), SM (41:2), SM (43:1), Cer (41:1) and Cer (43:1) by batch.

| **Phospholipid** | **Statistic** | **Batch 1** | **Batch 2** | **Batch 3** | **Batch 4** | **Batch 5** | **Batch 6** | **P-value** |
| --- | --- | --- | --- | --- | --- | --- | --- | --- |
| **SM 41:1** |  |  |  |  |  |  |  | <.0001 |
|  | **Mean** | 17.99 | 16.31 | 14.72 | 16.5 | 18.08 | 15.38 |  |
|  | **Standard Deviation** | 4.36 | 4.14 | 3.8 | 4.55 | 3.98 | 4.12 |  |
|  | **Median** | 18.05 | 16.6 | 14.1 | 16.25 | 17.9 | 14.8 |  |
|  | **25th percentile-75th percentile** | 14.95-21.15 | 13.50-18.20 | 12.50-16.50 | 13.30-19.30 | 15.60-20.80 | 11.70-18.50 |  |
| **SM 41:2** |  |  |  |  |  |  |  | <.0001 |
|  | **Mean** | 14.55 | 12.48 | 11.56 | 12.91 | 14.31 | 12.09 |  |
|  | **Standard Deviation** | 3.66 | 3.46 | 3.69 | 4.16 | 3.93 | 3.73 |  |
|  | **Median** | 13.9 | 11.9 | 11.05 | 12.75 | 13.7 | 11.7 |  |
|  | **25th percentile-75th percentile** | 12.20-16.85 | 9.93-15.00 | 8.61-14.10 | 9.85-14.80 | 11.50-16.10 | 8.84-15.00 |  |
| **SM 43:1** |  |  |  |  |  |  |  | <.0001 |
|  | **Mean** | 2.55 | 2.09 | 1.92 | 2.1 | 2.84 | 3.19 |  |
|  | **Standard Deviation** | 1.6 | 0.83 | 0.97 | 1.8 | 1.97 | 1.77 |  |
|  | **Median** | 2.05 | 2 | 1.76 | 1.44 | 2.13 | 2.96 |  |
|  | **25th percentile-75th percentile** | 1.40-3.13 | 1.65-2.39 | 1.24-2.23 | 1.11-2.35 | 1.55-3.31 | 2.07-3.94 |  |
| **Cer 41:1** |  |  |  |  |  |  |  | <.0001 |
|  | **Mean** | 0.64 | 0.38 | 0.57 | 0.71 | 0.62 | 0.52 |  |
|  | **Standard Deviation** | 0.21 | 0.12 | 0.3 | 0.24 | 0.18 | 0.14 |  |
|  | **Median** | 0.62 | 0.38 | 0.55 | 0.65 | 0.59 | 0.5 |  |
|  | **25th percentile-75th percentile** | 0.52-0.76 | 0.29-0.45 | 0.27-0.74 | 0.55-0.83 | 0.47-0.71 | 0.44-0.59 |  |
| **Cer 43:1** |  |  |  |  |  |  |  | <.0001 |
|  | **Mean** | 0.62 | 0.38 | 0.27 | 0.43 | 0.52 | 0.41 |  |
|  | **Standard Deviation** | 0.21 | 0.21 | 0.17 | 0.19 | 0.14 | 0.11 |  |
|  | **Median** | 0.64 | 0.38 | 0.21 | 0.39 | 0.5 | 0.39 |  |
|  | **25th percentile-75th percentile** | 0.50-0.75 | 0.23-0.46 | 0.16-0.35 | 0.32-0.52 | 0.42-0.64 | 0.33-0.46 |  |
| *not log-transformed or standardized |  |  |  |  |  |  |  |  |

**Supplementary Table 3:** PCA factors and their cross-sectional associations with grip strength, SPPB score, and 4 meter walking speed.

| **Physical Function Measure** | **Sphingolipid of Interest** | **Difference associated with 1 unit increase in Metabolite* (Model 1)** | **Difference associated with 1 unit increase in Metabolite** (Model 2)** | **Difference associated with 1 unit increase in Metabolite*** (Model 3)** |
| --- | --- | --- | --- | --- |
| **Grip strength (kg)** |  |  |  |  |
|  | Factor 1 | 0.20 (-0.70, 1.09) | -0.16 (-1.32, 0.98) | 0.62 (-0.62, 1.87) |
|  | Factor 2 | 0.59 (-0.20, 1.39) | 0.32 (-0.57, 1.20) | -0.14 (-1.08, 0.79) |
|  | Factor 3 | 0.23 (-1.29, 1.74) | 0.30 (-1.34, 1.95) | 0.004 (-1.35, 1.36) |
|  | Factor 4 | -1.04 (-2.72, 0.64) | -1.15 (-2.98, 0.69) | -0.88 (-2.41, 0.66) |
| **SPPB score (0-12 scale)** |  |  |  |  |
|  | Factor 1 | 0.32 (0.04, 0.61) | -0.19 (-0.53, 0.15) | 0.25 (-0.13, 0.62) |
|  | Factor 2 | 0.22 (-0.03, 0.47) | 0.14 (-0.12, 0.40) | -0.07 (-0.35, 0.21) |
|  | Factor 3 | 0.23 (-0.21, 0.68) | 0.10 (-0.47, 0.68) | 0.09 (-0.35, 0.53) |
|  | Factor 4 | -0.02 (-0.48, 0.44) | -0.40 (-0.95, 0.15) | -0.18 (-0.61, 0.26) |
| **4 meter walking speed (m/s)** |  |  |  |  |
|  | Factor 1 | 0.028 (0.003, 0.053) | -0.008 (-0.037, 0.023) | 0.005 (-0.028, 0.038) |
|  | Factor 2 | 0.014 (-0.008, 0.037) | 0.014 (-0.008, 0.037) | 0.010 (-0.015, 0.035) |
|  | Factor 3 | 0.028 (-0.009, 0.064) | 0.015 (-0.023, 0.051) | 0.013 (-0.022, 0.049) |
|  | Factor 4 | 0.024 (-0.014, 0.063) | -0.009 (-0.049, 0.032) | -0.008 (-0.048, 0.031) |
| *Adjusted for age, sex, race-site, and batch effects. | | | | |
| **Adjusted for model 1, plus body mass index, diabetes status, hypertension status, use of lipid lowering medication, total cholesterol, Digit Symbol Substitution test z-score, and previous stroke. | | | | |
| ***Adjusted for model 2, plus education, presence of an APOE-4 allele, Mini-mental state examination (MMSE) score, sports index, previous heart failure, previous coronary heart disease, CES depression score, HDL cholesterol, triglyceride levels, SF bodily pain score, current smoking status, and current drinking status. | | | | |

**Supplementary Figure 1.** Volcano plots of cross-sectional associations of sphingolipids with grip strength (A), SPPB score (B) and 4-meter walking speed (C) after covariate adjustments in Model 3 in ARIC visit 5, 2011-13. Model 3 adjusted for age, sex, race-site, and batch effects (variables in Model 1) body mass index, diabetes status, hypertension status, use of lipid lowering medication, total cholesterol, Digit Symbol Substitution test z-score, and prevalent stroke (variables in Model 2), and education, presence of an APOE4 allele, Mini-mental state examination (MMSE) score, sports index, previous heart failure, previous coronary heart disease, CES depression score, HDL cholesterol, triglyceride levels, SF bodily pain score, current smoking status, and current drinking status.


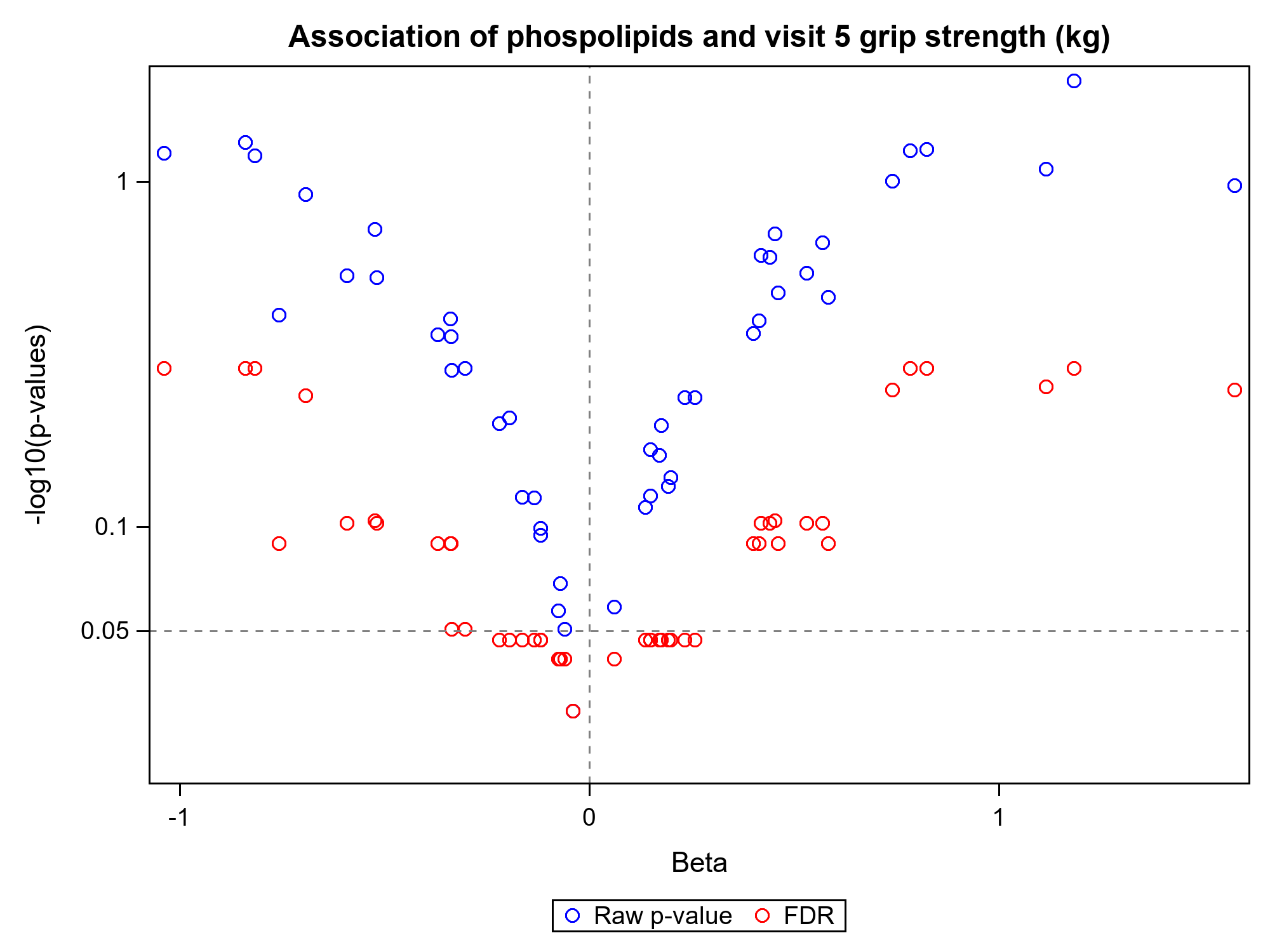
A

B


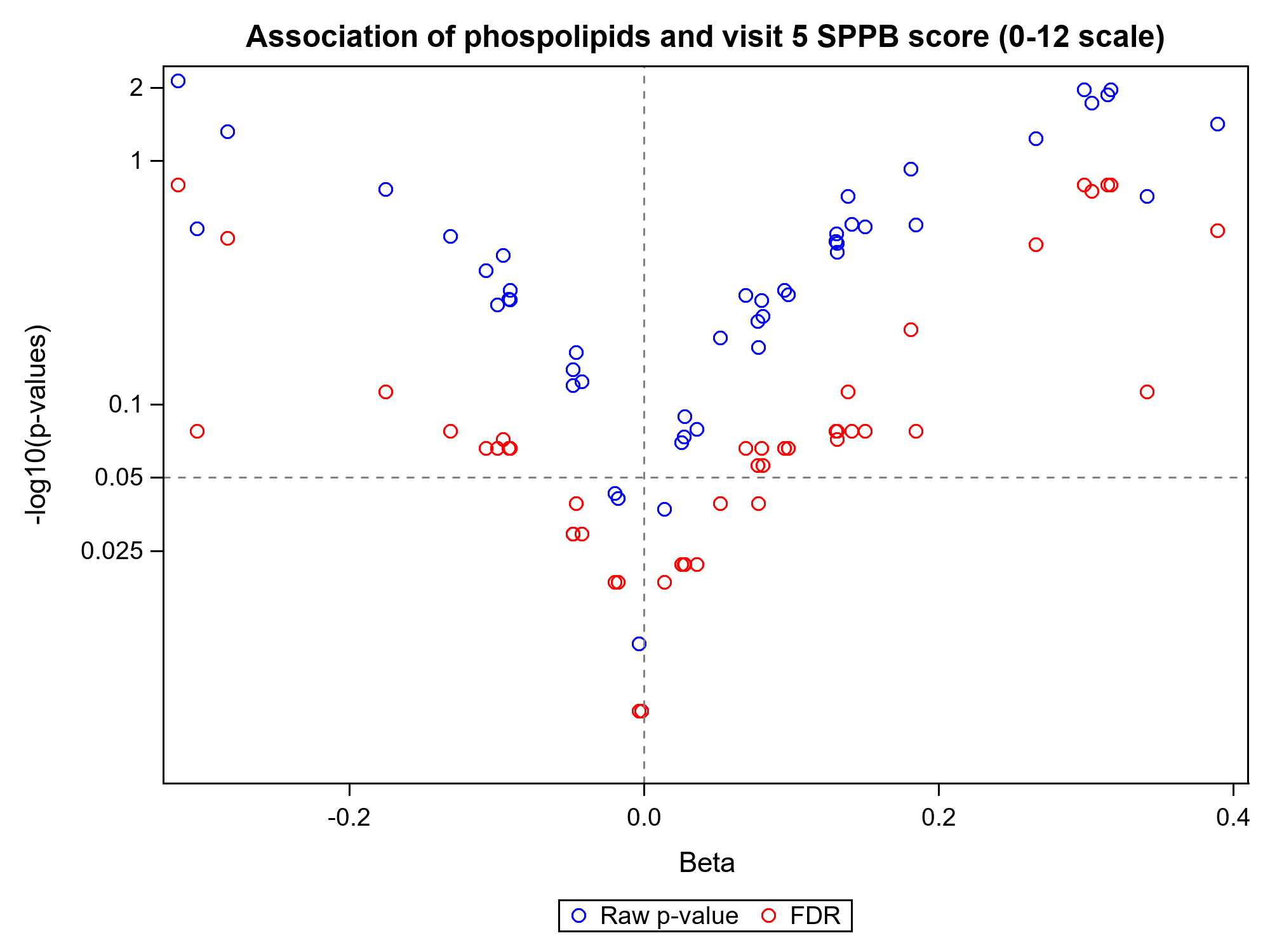


C


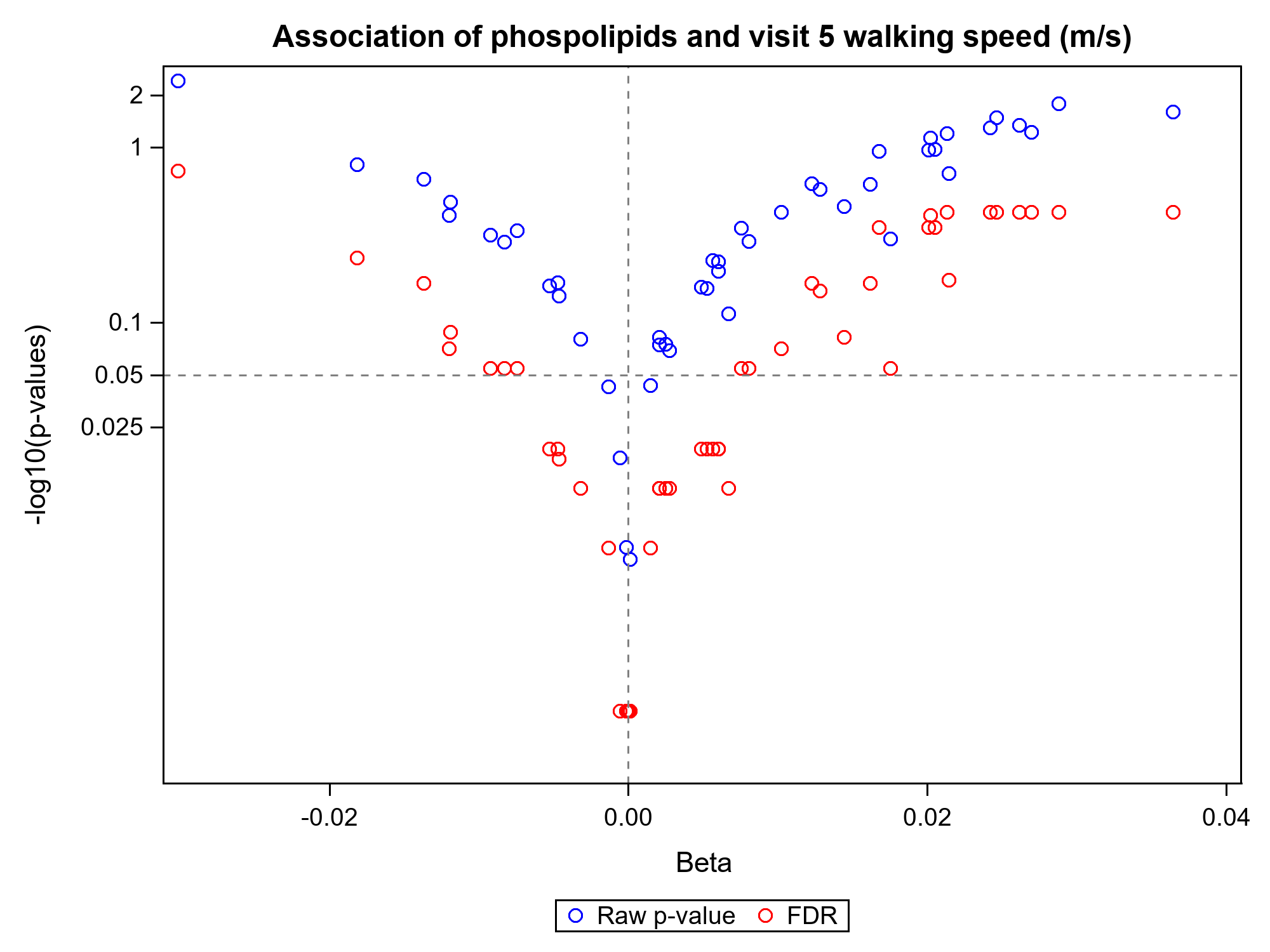


**Supplementary Figure 2.** Volcano plots of cross-sectional associations of sphingolipids with changes in grip strength (A), in SPPB score (B) and in 4-meter walking speed (C) after covariate adjustments in Model 3 between ARIC visits 5 and 6, 2011-17. Model 3 adjusted for age, sex, race-site, and batch effects (variables in Model 1) body mass index, diabetes status, hypertension status, use of lipid lowering medication, total cholesterol, Digit Symbol Substitution test z-score, and prevalent stroke (variables in Model 2), and education, presence of an APOE4 allele, Mini-mental state examination (MMSE) score, sports index, previous heart failure, previous coronary heart disease, CES depression score, HDL cholesterol, triglyceride levels, SF bodily pain score, current smoking status, and current drinking status.

A


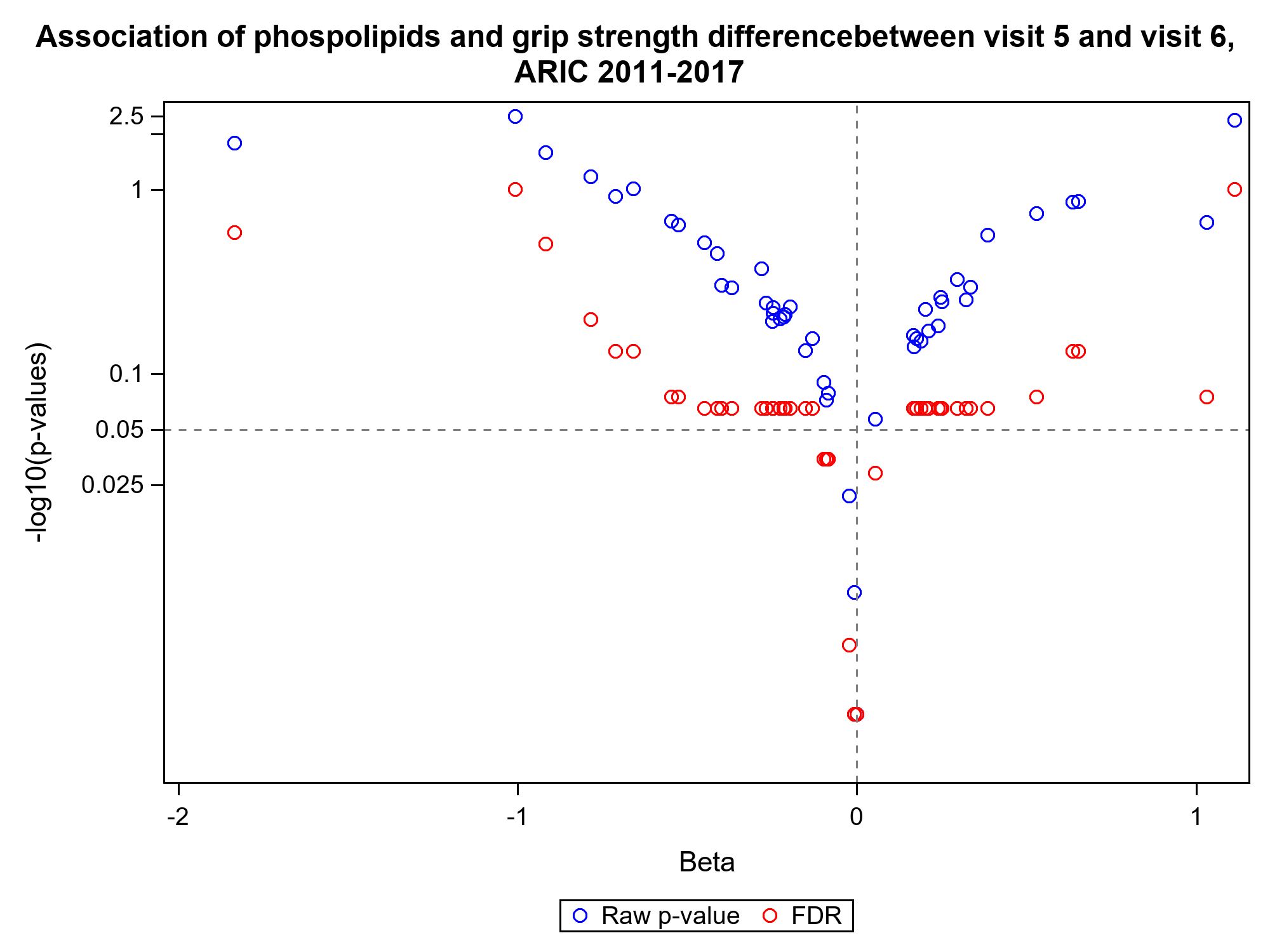


B


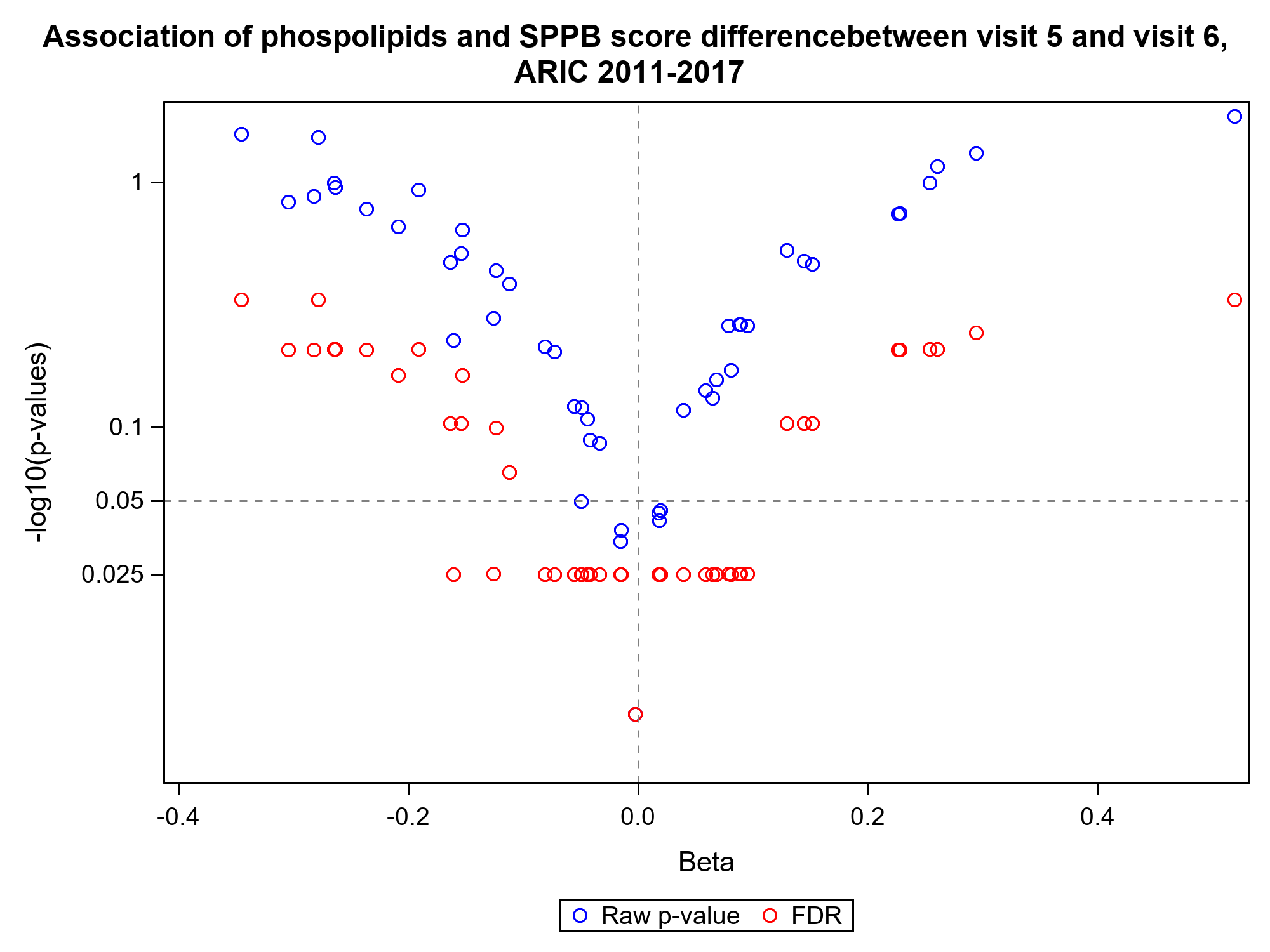


C


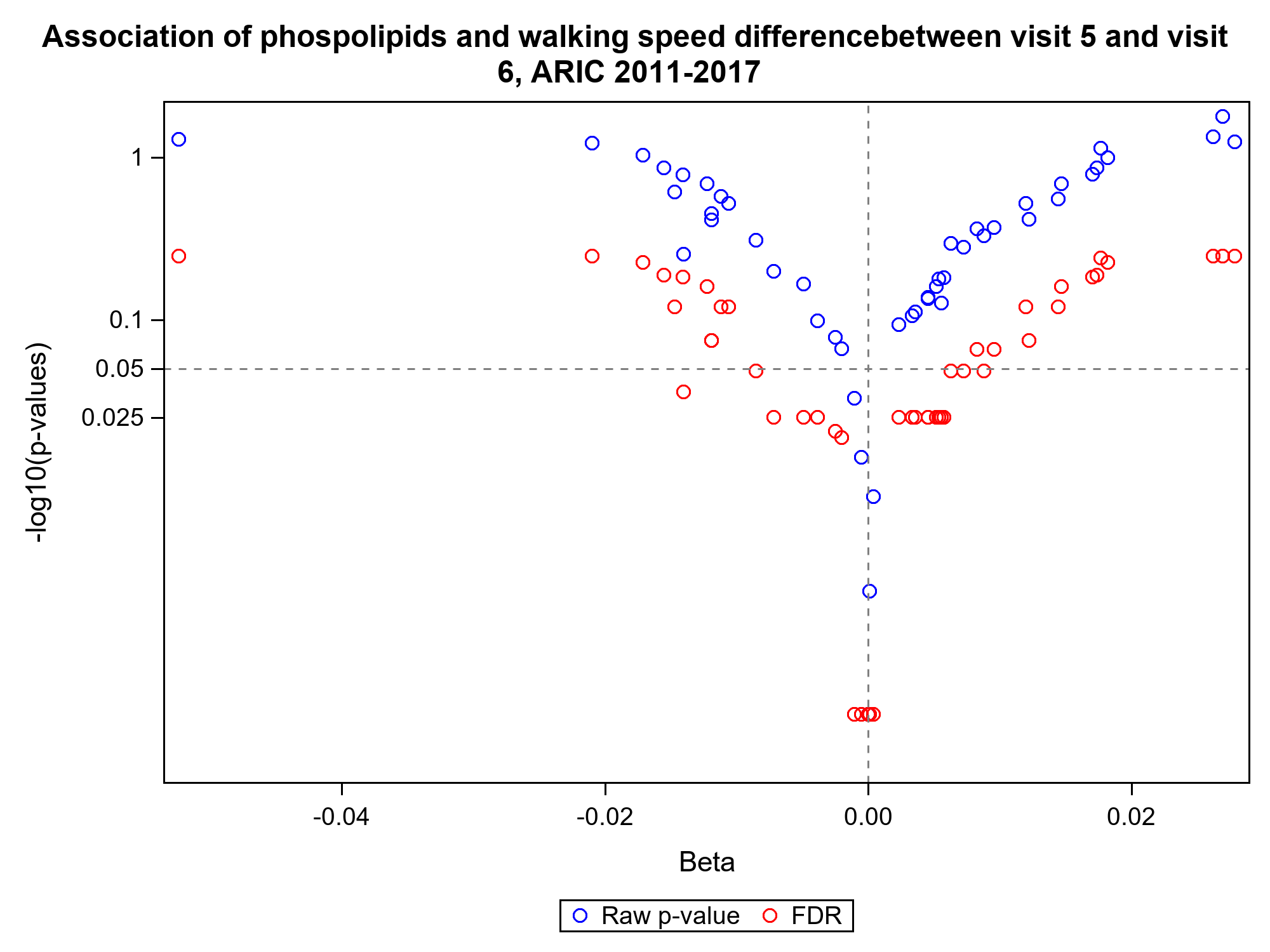

Supplement: Supplementary file 1 — Supplementary Information. [file 41598_2020_80929_MOESM1_ESM.docx]
